# Role of Endoplasmic Reticulum Stress in Atherosclerosis and Diabetic Macrovascular Complications

**DOI:** 10.1155/2014/610140

**Published:** 2014-07-01

**Authors:** Dmitry A. Chistiakov, Igor A. Sobenin, Alexander N. Orekhov, Yuri V. Bobryshev

**Affiliations:** ^1^Pirogov Russian State Medical University, Moscow, Russia; ^2^Institute for Atherosclerosis, Skolkovo Innovation Center, Moscow, Russia; ^3^Institute of General Pathology and Pathophysiology, Russian Academy of Sciences, Moscow, Russia; ^4^Russian Cardiology Research and Production Complex, Moscow, Russia; ^5^Faculty of Medicine and St. Vincent's Centre for Applied Medical Research, University of New South Wales, Sydney, NSW 2052, Australia; ^6^School of Medicine, University of Western Sydney, Campbelltown, NSW, Australia

## Abstract

Age-related changes in endoplasmic reticulum (ER) are associated with stress of this cell organelle. Unfolded protein response (UPR) is a normal physiological reaction of a cell in order to prevent accumulation of unfolded and misfolded proteins in the ER and improve the normal ER function. However, in pathologic conditions such as atherosclerosis, obesity, and diabetes, ER function becomes impaired, leading to the development of ER stress. In chronic ER stress, defective posttranslational protein folding results in deposits of aberrantly folded proteins in the ER and the induction of cell apoptosis mediated by UPR sensors C/EBP*α*-homologous protein (CHOP) and inositol requiring protein-1 (IRE1). Since ER stress and ER-induced cell death play a nonredundant role in the pathogenesis of atherosclerosis and diabetic macrovascular complications, pharmaceutical targeting of ER stress components and pathways may be beneficial in the treatment and prevention of cardiovascular pathology.

## 1. Introduction

The endoplasmic reticulum (ER) is a complex cytoplasmic membrane structure presented in eukaryotic cells. ER is involved in protein folding, lipid synthesis, and regulation of the intracellular calcium balance [1]. Secretory and membrane proteins, which are synthesized in ER, undergo proper folding in the ER lumen. ER chaperones such as glucose-regulated protein 78 kDa (GRP78 or BiP) and GRP94, oxidoreductases, and high calcium concentrations are essential for proper protein folding and assembling [2]. GRP78 is a Ca^2+^-dependent chaperone that is responsible for the folding of hydrophobic protein regions [3]. Protein disulfide isomerase (PDI) is involved in the formation of disulfide bonds whereas ER thiol oxidase (ERO1) initiates disulfide transfer to oxidized proteins [4]. Aging-related changes in ER are associated with stress of this cell organelle [5]. The oxidative protein folding is associated with advanced production of reactive oxygen species (ROS) that may lead to extensive oxidative stress and cell apoptosis [6]. Indeed, the ER is vulnerable to various stressors capable of disturbing the redox homeostasis in the ER lumen.

## 2. Unfolded Protein Response

**Figure 1:**
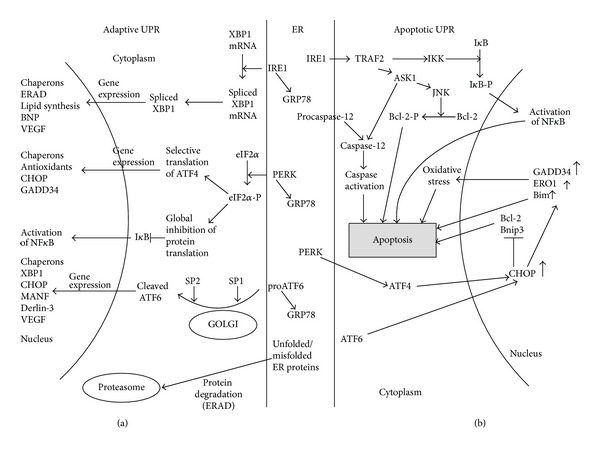
The adaptive and proapoptotic UPR pathways. (a) Adaptive UPR mechanism. In nonstressed conditions, the ER chaperone GRP78 binds to all three ER stress sensors such as PERK, IRE1, and ATF6. In ER stress, GRP78 dissociates from the ER sensors, and this leads to their activation. eIF2*α* is phosphorylated by PERK and dephosphorylated by GADD34. Phosphorylated eIF2*α* blocks global protein translation but remains selective translation of several proteins including transcriptional factor ATF4. ATF4 then initiates expression of UPR-related genes. Upon activation, ATF6 translocates from the ER to the Golgi complex where it is cleaved by proteases S1P and S2P. Cleaved ATF6 acts as a transcriptional factor activating expression of several UPR- and non-UPR genes including XBP1. Activated IRE1 specifically splices XBP1 mRNA. Spliced XBP1 shows transcription factor activity to induce UPR- and non-UPR genes. Proteasome plays an important role in degradation of unfolded and misfolded proteins. Thus, production of proteasome components is also stimulated to increase utilization of misfolded proteins through the mechanism of ERAD. (b) Proapoptotic UPR mechanism. The apoptotic pathway is induced in chronic and prolonged ER stress. CHOP plays a key role in mediating ER stress-induced apoptosis. CHOP expression is stimulated by ATF4- and ATF6. CHOP represses expression of antiapoptotic proteins Bcl-2 and Bnip3 and activates translocation of proapoptotic protein Bim to the ER membrane. IRE1*α* forms a complex with the adaptor protein TRAF2, which consequently activates ASK1 and JNK. Activation of JNK induces apoptosis cell through phosphorylation of several Bcl-2 family members. The IRE1*α*/TRAF2 complex also binds to I*κ*B kinase, and this results in activation of transcription factor NF-*κ*B. Prolonged ER stress activates caspase 12 that in turn activates caspase-9/3 thereby leading to the mitochondria-independent apoptotic pathway.

Incompletely folded or misfolded proteins are subjected to ER-associated degradation (ERAD) that occurs in cytoplasm. ER-mediated protein synthesis and folding are strictly regulated. Impairments in ER folding capacity may result in the accumulation of unfolded proteins and induce ER stress. In the ER, three proteins are able to sense increase in misfolded polypeptides and initiate the unfolded protein response (UPR). The UPR sensors include activating transcription factor-6 (ATF6), inositol requiring protein-1 (IRE1), and protein kinase RNA-like ER kinase (PERK). All three proteins have domains exposed to the ER lumen and are capable of binding GRP78 [7]. In normal conditions, GRP78 is bound to the molecules of UPR sensors. In ER stress, GRP78 dissociates from the UPR sensors that leads in turn to induction of UPR (Figure 1).

The UPR applies several mechanisms to minimize ER stress. One of those mechanisms involves the activation of chaperone synthesis in order to improve and intensify the intraluminal protein folding. Another mechanism includes protein translation arrest in order to prevent further protein load into the ER [26]. The ER folding capacity may be also improved indirectly, through stimulating ER biogenesis [6]. In a case of chronic long-term or acute ER stress, when the adaptive UPR is unable to stop the stress, apoptotic pathways are activated in the stressed cell [27]. ER sensor proteins such as IRE1 and PERK are involved in both the adaptive and the proapoptotic UPR pathways.

### 2.1. IRE1

Among ER stress sensors, IRE1 is the most evolutionarily preserved. In normal conditions, IRE1 and GRP78 interact with each other, and this prevents IRE1 activation [6]. In ER stress, GRP78 becomes released from the complex with IRE1. IRE1 is then activated by self-phosphorylation. The active IRE1 is able to specifically splice mRNA encoding X-box binding protein (XBP)1 thereby inducing translation of functionally active XBP1 [1]. XBP1 induces transcription of chaperones and other UPR-related proteins and enhances the degradation of misfolded proteins [28]. By degrading mRNAs other than XBP1, IRE1 contributes to reducing protein load to the ER [29].

However, in long-lasting ER stress, IRE1 can be involved in activation of proinflammatory pathways and apoptosis. IRE1 forms a complex with the adaptor protein tumor necrosis factor (TNF) receptor-associated factor (TRAF)2 [30] that in turn recruits mitogen-activated protein kinase, apoptosis signal-regulating kinase (ASK) [31], and caspase 12 [32]. The complex activates I*κ*B kinase followed by I*κ*B kinase-mediated suppression of the inhibitor of *κ*B protein and induction of the nuclear factor (NF)-*κ*B. Since NF-*κ*B controls expression of many proinflammatory genes, IRE1 is therefore suggested to provide a link between the ER stress and inflammation [33].

### 2.2. PERK

This ER stress sensor molecule belongs to the family of serine threonine kinase and has a high degree of homology with ERE1. Both IRE1 and PERK share similar mechanisms of activation involving dissociation of GRP78 from the luminal binding domain and self-phosphorylation upon stress conditions. After activation, PERK downregulates eukaryotic translation initiation factor 2*α* (eIF2*α*) that is needed for cap recognition and therefore is essential for further induction of cap-dependent transcription. eIF2*α* inactivation results in marked decrease of protein load to the ER [6]. Interestingly, phosphorylated eIF2*α* is responsible for translation of several mRNAs including mRNA for transcriptional factor ATF4. This factor is responsible for the induction of the negative feedback regulatory loop since it activates expression of GADD34, a regulatory subunit of the phosphatase that dephosphorylates eIF2*α* and restores cap-dependent translation [34]. Indeed, ATF4 regulates protein translation during ER stress.

ATF4 stimulates expression of C/EBP*α*-homologous protein (CHOP, or GADD153). CHOP expression can be also induced by ATF6 and XBP1, but the PERK-eIF2*α*-dependent pathway is prevalent [35]. CHOP is a transcription factor that induces apoptosis through several mechanisms including upregulation of ERO1*α*, which then mediates Ca^2+^-dependent apoptotic pathway, and downregulation of antiapoptotic factors Bcl-2 and Bnip3 [36, 37].

ERO1*α* is involved in reoxidation of PDI yielding production of hydrogen peroxide, a byproduct of disulfide bond formation [38]. Therefore, ER stress-induced upregulation of ERO1*α* may lead to ROS overproduction and advanced oxidative stress that in turn contribute to cell apoptosis [5]. ERO1*α* activation stimulates inositol-1,4,5-trisphosphate receptor-1 (IP3R1), a ER-associated Ca^2+^ channel [39] that triggers depletion of the intraluminal calcium reservoir. Increase in cytoplasmic Ca^2+^ promotes stimulation of calcium/calmodulin-dependent protein kinase II, which plays a key role in induction of several proapoptotic pathways including activation of the death receptor FAS and mitochondrial release of apoptogens [40]. CHOP is directly involved in induction of expression and translocation to the ER membrane of the proapoptotic protein Bim [41].

### 2.3. ATF6

Upon initiation of ER stress, ATF6 is cleaved by two (site 1 and site 2) proteases associated with the Golgi complex. After cleavage, the cytosolic N-domain of ATF6 translocates into the nucleus where it triggers expression of many UPR-related genes including GRP78 and XBP1 [26]. ATF6 activates expression of Derlin-3 that enhances the ERAD activity [42]. Before degradation by the proteasome, most of misfolded proteins are ubiquitinated and extracted by the cytosolic ATPase p97 [43, 44].

## 3. Role of ER Stress in Atherosclerosis

Prolonged ER stress observed in atherosclerotic lesions is an important contributor to proatherogenic progression [45]. ER stress was found in all major cell type in atherosclerosis including macrophages, vascular smooth muscle cells (VSMCs), and endothelial cells (ECs).

### 3.1. ER Stress in Macrophages

**Figure 2:**
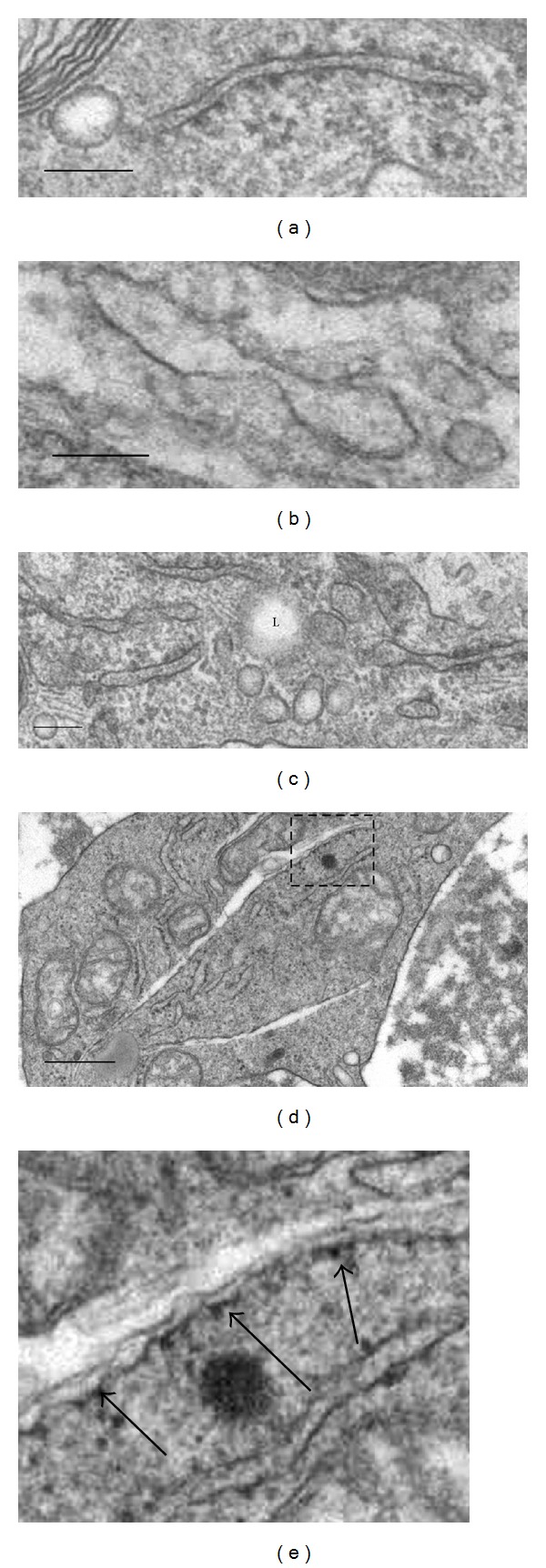
Structural alterations of cisterns of granular endoplasmic reticulum (ER) in macrophages residing in human atherosclerotic lesions (identified by means of electron microscopy) (a–e). In contrast to intact ER cistern appearance (a), some ER cisterns display a notable expansion of the intracisternal space (b) and demonstrate focal disappearance of ribosomes from the internal membranes of cisterns (a, b). In some macrophages, the expansion of the intracisternal space is accompanied by degenerative alterations of ER cistern (d, e). (e) is a detail of (d). The arrows in (e) show ribosomes which are still present on the internal surface of a degenerating ER cistern. In (c), L: lipid droplet. Bars = 100 nm (a–c), 500 nm (d).

In normal macrophages, low density lipoprotein (LDL) cholesterol particles are loaded from late endosomes to the ER. In the ER, cholesterol is esterified and accumulates to form inert lipid droplets [46]. In atherosclerotic macrophages, ER-mediated cholesterol reesterification is markedly reduced or failed resulting in heavy intracellular deposits of nonesterified cholesterol in foam cells [47]. Electron microscopy observations revealed that ER in atherosclerotic macrophages undergoes a remarkable change (Figure 2). In foam cells, intraluminal ER oxidoreductases oxidize cholesterol to 7-ketocholesterol (7-KC) and other oxysterols. Oxysterols are highly cytotoxic and may induce cell death through ROS-mediated oxidative damage and other mechanisms [48].

Prolonged ER stress contributes to apoptosis of lesional macrophages. Apoptosis associated with robust expression of CHOP was shown in human lesions [45] and atherosclerotic plaques of apolipoprotein (apo)E-deficient mice [49]. Inactivating Chop in apoE-deficient mice leads to decreased rates of macrophage apoptosis and plaque necrosis [50, 51]. CHOP contributes to ER stress-induced macrophage death by inducing Fas activation, depletion of ER-associated calcium stores, and release of apoptogens from mitochondria [52].

In early plaques, apoptotic cells are quickly phagocytized by macrophages [53]. This process is driven by anti-inflammatory cytokines such as transforming growth factor- (TGF-) *β* and interleukin- (IL-) 10 [54]. In advanced plaques, macrophages cannot efficiently clear dying cells that become necrotic [55]. This results in the formation of the inflammatory necrotic core [56].

In some circumstances, ER stress alone is not strong enough to induce apoptosis in macrophages. Additional stimuli such as the activation of pattern recognition receptors (PRRs) are required to initiate cell death [56]. PPRs include various Toll-like receptors (TLRs) and scavenger receptors. In plaque macrophages, PPRs may be activated by oxidized lipids, and this leads to the induction of apoptosis via CD36-TLR2 pathway and is accompanied with NADPH oxidase-mediated oxidative stress [57]. NADPH oxidase contains a subunit Nox2 whose inhibition minimizes ER stress-induced macrophage death [58]. These findings suggest a central role of this enzyme as a link between the oxidative stress and ER stress in promoting macrophage apoptosis. In addition, upregulation of NADPH oxidase further aggravates apoptotic process through stimulating PERK-CHOP-dependent mechanism.

At low doses, ER stress inducers such as modified (oxidized and acetylated) LDL, 7-KC, and peroxynitrite donor SIN are able to stimulate macrophage PRRs and cause NADPH oxidase-mediated ROS production [56, 59]. In a “two-hit” hypothesis, ER stress in plaque macrophages should be induced by a low-dose ER stressor such as PRR ligands that in turn triggers macrophage apoptosis [57, 59].

Lipoprotein(a) [Lp(a)], an LDL-like lipoprotein, and oxidized phospholipids are established to represent strong risk factors for human atherosclerosis [59]. To initiate apoptosis in ER-stressed macrophages, both atherogenic lipid inducers utilize similar mechanisms involving the activation of CD36-TLR2 signaling and oxidative stress [57].

Lp(a) is a major carrier of oxidized phospholipids in human blood [60]. According to the “two-hit” hypothesis, Lp(a) could therefore mediate apoptosis in human plaque macrophages.

### 3.2. ER Stress in Endothelial Cells

In EC, various ER stress inducers were shown to initiate UPR. For example, shear stress activates IRE1-dependent UPR [61, 62] whereas oxidized phospholipids and homocysteine induce both IRE1- and CHOP-mediated pathways [63–65]. In dynamic models of shear stress, a variety of UPR-related molecules including ATF6, GRP78, IRE1, and XBP1 were upregulated in ECs [61, 62, 66, 67]. XBP1 is always overexpressed in advanced plaques, a finding that may reflect a proatherogenic role of long-term XBP1 upregulation whereas limited stimulation of this ER stress effector may be protective against ER stress [61].

ER stress induced by modified (oxidized and glycated) LDL results in the development of oxidative stress and oxidation-mediated inhibition of sarcoplasmic/endoplasmic reticulum Ca^2+^-dependent ATPase (SERCA), a calcium pomp resided in the ER [8]. AMP kinase (AMPK) *α*2 suppresses SERCA oxidation, and inhibition of this kinase in LDL receptor- (Ldlr-) deficient mice leads to advanced ER stress and atherogenesis [9]. Thus, alterations in calcium homeostasis caused by oxidative stress play a crucial role in ER stress-mediated endothelial dysfunction in atherosclerotic vessels. ER stress-induced apoptosis diminishes the barrier function of the vascular endothelium and induces procoagulant phenotypic changes in ECs that may be directly responsible for increased risk of thrombosis and other late atherosclerotic complications [68].

### 3.3. ER Stress in VSMCs

A stable plaque phenotype may be critically disturbed by apoptosis in VSMCs that alters the formation of the protective fibrous cap [69]. In VSMCs, CHOP-mediated apoptotic mechanism may be induced by numerous ER stressors such as 7-KC, homocysteine, glucosamine, free cholesterol, and others [70–74]. CHOP-dependent apoptosis is accompanied with enhanced formation and release of ROS, and N-acetylcysteine, an anti-oxidant, may therefore protect cultured VSMCs against apoptotic death [73].

Elevated plasma levels of homocysteine are considered to increase atherosclerotic risk in humans and animal models [71, 75]. Hyperhomocysteinemia is believed to induce ER stress through alterations of calcium balance [76] and upregulation of sterol response element binding protein-2 (SREBP-2) that increases lipid deposits in VSMCs [77, 78]. Glucosamine that accumulates in vascular cells in diabetes may have a primary responsibility for ER stress induction in VSMCs of diabetic patients associated with GRP78 upregulation [74]. However, the mechanisms of ER stress-mediated apoptosis in VSMCs are significantly less studied compared to those of macrophages and ECs.

## 4. ER Stress and Obesity

The human body is able to accumulate extra fat in the adipose tissue to survive in starvation. Normally, fat is deposited in adipocytes. However, regular intake of fat-rich diet and alterations in lipid metabolism may lead to the phenomenon of ectopic fat storage, when fat accumulates not only in adipocytes but also in nonadipocyte cells. In obesity, higher free fatty acids levels may enhance lipid accumulation in macrophages and promote formation of foam cells [79].

In obese people, adipocytes are particularly vulnerable to ER stress and apoptosis due to abnormal fat deposits and upregulated lipid metabolism [80]. Macrophages resided in the adipose tissue phagocytize both the extra fat droplets and apoptotic adipocytes releasing high amounts of ROS by mitochondria. Excessive ROS production drives further progression of cellular stress and increases secretion of adipokines in adipocytes [81]. Adipokines promote preferential differentiation of macrophages towards the proinflammatory M1 phenotype [82].

Adiposity is associated with enhanced M1 macrophage-dependent production of multiple proinflammatory mediators such as IL-1*β*, IL-6, TNF-*α*, and CXCL10. M1 macrophages inhibit adipocyte hypertrophy and adipogenesis [83] and support low-grade inflammation in the adipose and nonadipose tissues including vessels [84]. In lesional macrophages, adiposity promotes ER stress by activation of the macrophage fatty acid-binding protein-4, also known as adipocyte fatty acid binding protein aP2 that mediates transfer of saturated fatty acids (SFAs) [19]. Increase in SFA levels leads to the induction of apoptosis in macrophages. ApoE-deficient mice lacking aP2 have reduced atherosclerotic lesions, in which expression of XBP1 and PERK is downregulated and macrophage apoptosis is decreased [85]. Inactivation of aP2 protects macrophages from palmitate-induced ER stress and apoptosis [19]. In aP2-deficient macrophages, expression of transcription factor LXR*α* is activated, and this factor stimulates transcription of stearoyl-CoA-desaturase 1, an enzyme converting SFAs to monounsaturated fatty acids, which are significantly less potent of inducing ER stress [86]. Indeed, activation of LXR*α* in aP2-deficient macrophages prevents ER stress while overexpression of aP2 in macrophages and adipocytes, in contrast, supports ER stress induction and atherogenesis.

This protective effect is mediated by increased expression of transcription factor LXR*α* in aP2-deficient macrophages. This factor activates expression of stearoyl-CoA-desaturase 1, converting SFAs to monounsaturated fatty acids that are significantly less capable of inducing ER stress [86].

## 5. ER Stress and Diabetes 

### 5.1. Insulin Resistance-Induced ER Stress in Macrophages

In diabetic subjects with atherosclerosis, the proatherogenic role of ER stress and CHOP-mediated macrophage apoptosis is significantly enhanced that results in the development of advanced plaques with the especially large necrotic core [87, 88]. Macrophages were shown to have functional insulin receptors, and insulin resistance (IR) is a potent inducer of chronic ER stress in macrophages [89]. High insulin doses suppress insulin signaling in macrophages [88]. Under diabetic conditions, insulin signal transduction in macrophages is also inhibited by diacylglycerol-dependent activation of protein kinase C [90].

Expression of the scavenger receptor SRA is markedly upregulated in IR macrophages. Indeed, according to the “two-hit” hypothesis, these macrophages should be particularly sensitive to PRR-driven ER stress and apoptosis [91]. Experiments with cultured IR macrophages loaded with lipoprotein-derived free cholesterol do show markedly increased apoptosis that suggest a key role of SRA-induced mechanism of ER stress in mediating death of IR macrophages [92]. In these macrophages, MEK/ERK/cAMP-responsive element-binding protein (CREBP) signaling and calcium homeostasis are disturbed [89]. Alterations in intracellular calcium balance involve depletion of ER calcium stores and reduced SERCA activity. Antidiabetic agent exenatide rescues IR macrophages from apoptosis by activation of the macrophage glucagon-like peptide 1 (GLP-1) receptor followed by restoring MEK/ERK signaling and inhibition of Ca^2+^-dependent apoptosis [89].

In IR and ER-stressed macrophages, activity of the serine/threonine-specific protein kinase Akt is lowered and Akt- and NF-*κ*B-dependent pathways responsible for cell survival are suppressed [92]. Insufficient Akt activity in IR macrophages is associated with preferential localization of transcription factor FoxO1 in the nucleus [93]. Normally, Akt-dependent phosphorylation of FoxO1 in response to insulin signaling initiates translocation of this factor to cytoplasm where it is inactivated by proteolytic degradation. Macrophages deficient for FoxO1, 3, and 4 are resistant to ER stress-driven apoptosis [93]. The preferential nuclear location of FoxO1 correlates with enhanced expression of I*κ*B*ε*, an inhibitor of NF-*κ*B, which in turn increases apoptosis of IR macrophages [94].

### 5.2. Glucosamine-Induced ER Stress

Diabetic hyperglycemia significantly increases cardiovascular risk inducing vascular dysfunction through inhibitory effects on proliferation of vascular cells and enhancement of their apoptosis [95–98]. Several pathologic mechanisms link diabetic hyperglycemia and atherosclerosis. Activation of the aldose reductase pathway alters redox homeostasis and promotes oxidative stress-mediated damage of vascular cells [99]. High glucose induces overactivity of protein kinase C that leads to reduced endothelial vasodilation [100] and increased ROS production [101]. Nonenzymatic glycation is markedly increased in diabetic patients, and this results in uncontrolled production of advanced glycation end-products (AGEs) [102] whose accumulation in blood plasma is related to enhanced modification of lipoproteins thereby increasing their atherogenicity [103]. Receptor for AGE (RAGE) is expressed in macrophages, ECs, and VSMCs [104], and AGE-RAGE interaction induces signaling pathways associated with increased ROS production and activation of inflammatory response in vascular cells [105].

In the hexosamine pathway, glutamine: fructose-6 phosphate amidotransferase (GFAT) catalyzes conversion of glucose to glucosamine-6 phosphate (G-6P) [106]. Diabetic hyperglycemia activates the hexosamine pathway that leads to the production of elevated G-6P levels in vascular cells [107, 108]. UDP-N-acetylglucosamine (UDP-GlcNAc), an end-product of the hexosamine pathway, is involved in both O- and N-linked protein glycation. N-glycosylation is an important stage of posttranslational modifications of newly synthesized proteins performed in the ER lumen [109]. Inhibition of N-glycosylation by tunicamycin (UDP-GlcNAc antagonist) activates the UPR [110].

GFAT is a rate-limiting enzyme in the hexosamine pathway. Overactivity of this enzyme in diabetic conditions promotes ER stress via stimulation of expression of UPR-related genes and contributes to downstream events including lipid accumulation and activation of proinflammatory and apoptotic pathways [111]. In contrast, GFAT inhibition attenuates ER stress [74]. Cultured human aortic VSMCs and macrophages treated with glucosamine develop apoptosis [74, 112, 113]. Therefore, suppression of GFAT could have a therapeutic potential in prevention of glucosamine-induced ER stress and apoptosis.

Glycogen synthase kinase (GSK)-3 whose expression is activated in glucosamine-induced ER stress may represent another potential target for antiatherogenic therapy [114]. GSK-3*β* activation in the aorta apoE-deficient hyperglycemic hyperhomocysteinemic mice fed on high-fat diet correlates with advanced atherosclerosis [115]. GSK-3*α* and *β* are two enzyme isoforms implicated in a variety of signaling pathways [116]. Upon the UPR induction, the inactive enzyme phosphorylated at Ser(21/9) is rapidly degraded in lysosomes that yields increase in GSK-3 activity [117]. Inhibition of GSK-3 displays both atheroprotective and anti-ER stress effects in cell cultures [118, 119] and hyperglycemic murine models [120].

## 6. Therapeutic Targeting of UPR Components and Its Clinical Potential

Targeting of proteins in ER stress and ER stress-induced apoptosis may be of high therapeutic value for treatment of human diseases in which ER stress plays a substantial role (Table 1). Promoters of GRP78 and GRP94 genes share significant sequence homology that explains the high concordance in coordinated expression of both ER chaperones [121]. Activation of ER chaperones plays an important role in adaptive UPR since it improves protein folding and prevents ER stress-induced apoptosis. Overexpression or stimulation of GRP78/94 had beneficial effect on ER-stressed cardiomyocytes [122–124] and showed cardioprotective properties in experiments* in vivo* [11–13].

Chemical chaperones such as phenylbutyrate and tauroursodeoxycholic acid (TUDCA) are able to stabilize proteins in their native conformation thereby mimicking properties of native ER chaperones [125]. Murine macrophages and adipocytes treated with chemical chaperones showed resistance to ER stress [19]. At present, phenylbutyric acid (PBA) in its sodium salt form is approved for therapy of urea cycle disorders [126] and is in process of clinical testing for treatment of some genetic disorders related to protein misfolding [127, 128]. PBA was shown to reduce ER stress and normalize glucose levels in diabetic mice [129]. Taking into account clinical approval of PBA for therapy of several diseases, it would be interesting to check whether PBA is efficient for treatment of cardiovascular pathology.

TUDCA was shown to display antiapoptotic and anti-ER stress properties for many types of cells and many diseases including atherosclerosis. TUDCA was able to block ER stress and slow lesion progression in Ldlr-deficient mice [9] and efficiently prevent apoptosis and ER stress induced by oxidized LDL in murine macrophages transgenic for human APOE4, a genetic risk variant for Alzheimer disease and atherosclerosis [130]. The antiapoptotic function of TUDCA can be released through restoring calcium homeostasis and SERCA activity [131] and downregulation of proapoptotic protein Bad [132].

Salubrinal specifically inhibits eIF2*α* phosphatases [23] and therefore supports blocking protein synthesis mediated by phosphorylated eIF2*α* [133]. Salubrinal is able to stop ER stress-induced apoptosis by inhibiting synthesis of members of proapoptotic signaling such as CHOP and caspase-12 in cardiac myocytes [134] and upregulating GRP78 in neurons [23]. However, in pancreatic *β*-cells, salubrinal induced activation of ATF4-CHOP mechanism that resulted in severe ER stress and apoptosis [133]. Thus, various cell types differently respond to salubrinal, and this limits its utility as a broad spectrum antiapoptotic drug [135].

CHOP is crucial in inducing ER stress-mediated apoptosis and hence development of CHOP inhibitors would be beneficial in prevention of atherosclerosis and treatment of heart failure and cardiac hypertrophy [50]. To date, no pharmacological agents specific for CHOP are available but there are drugs able to target molecular components of CHOP-mediated signaling. For example, SB203580, an inhibitor of p38 mitogen-activated protein kinase disrupts CHOP phosphorylation [136]. Mitogen-activated protein kinase blockers indirectly inhibit CHOP-dependent signaling in ER stress-induced apoptosis. JNK inhibitor SP600125 showed ability to suppress mechanical stretch-induced activation of CHOP [24].

Inhibition of ERO1*α* results in disruption of ER stress induced by oxidative stress and CHOP. Furthermore, several selective ERO1*α* inhibitors were developed. The inhibitor EN460 inactivates ERO1*α* by blocking its reoxidation [15]. Inhibitors EN460 and QM295 are able to launch the adaptive UPR signaling that prevents ER stress [15, 16]. Advanced ROS production induced by ERO1*α* overactivation may be efficiently suppressed by the antioxidant N-acetylcysteine [137] and by the treatment with curcumin and masoprocol that protect PDI from oxidative inactivation [138].

Restoring proteasome function, which is inhibited in ER stress [139], by protein kinase A activators such as isoproterenol or forskolin helps to attenuate ER stress-induced apoptosis [17]. TNF-*α* is significantly upregulated in ER stress, and inhibition of this cytokine by pravastatin [22] or TNF-*α*-specific antibody [24] results in significant protection of cardiomyocytes and other cells against apoptotic death. Hyperactivity of ASK1, a downstream target of IRE1-mediated signaling, contributes to the development of cardiac hypertrophy and heart failure, and inhibition of ASK1 by benzodiazepinones may be helpful for therapy of these cardiopathies [140, 141].

AMPK regulates switching from anabolic pathways (fatty acid synthesis, protein synthesis, etc.) to catabolism (fatty acid oxidation, glucose transport, etc.) thereby elevating energy levels in the cell [142]. The RNAse activity of IRE1 is probably required to activate AMPK that leads to the induction of the proper UPR and increases cell survival [143]. AMPK activation has the cardioprotective effect through reducing cardiac ER stress [10, 144]. Inactivation of AMPK is associated with severe ER stress and atherosclerosis that can be reduced by ER stress suppressors such as tempol or TUDCA [8, 9]. In contrast, multiple AMPK agonists such as 5-aminoimidazole-4-carboxyamide-1-*β*-D-ribofuranoside (AICAR), atorvastatin, A-769662, and PT1 reduce cardiovascular disease by blocking ER stress [10, 145]. Currently, AMPK activators are implicated in the treatment of obesity and metabolic syndrome. However, these drugs may be very helpful in antiatherogenic and cardioprotective therapy [146].

## 7. Conclusion: Limitations and Challenges in Anti-ER Stress Therapy

The UPR can be targeted by two ways including the activation of components of the adaptive mechanism of UPR and inhibition of those involved in the proapoptotic pathways of UPR. However, several questions should be answered to increase our understanding of mechanisms by which UPR targeting may help in therapy of cardiovascular disease. For example, one ER stressor (ATF6) has a cardioprotective role [147, 148] while two others (IRE1*α* and PERK) are involved in both the adaptive and proapoptotic UPR pathways. To date, the mechanisms controlling the switch from cell survival to death are not fully understood. Indeed, we do not know precisely when to activate or inhibit ER stress sensor proteins for treatment.

A variety of chemical inhibitors of protein kinases including receptor tyrosine kinase inhibitors are available. Some of those including sunitinib can directly activate IRE1 that results in XBP1 splicing and decreased ER stress [25]. Sunitinib malate is approved for use in treating renal cell carcinoma and gastrointestinal stromal tumor. However, in patients with a previous history of hypertension and coronary heart disease, sunitinib increases risk for cardiovascular events [149]. Thus, kinase inhibitors especially those that have a broad target spectrum should be carefully evaluated to prevent acute side effects.

In preclinical studies, chemical chaperones showed promising results in the improvement of ER folding capacity [125]. However, there are some limitations that seriously affect the therapeutic efficiency of these agents. Typically, high doses of these small molecule drugs are required to reach the desired effect. In addition, the UPR components are broadly expressed and their inhibition/activation in one tissue or organ may negatively influence the function of another tissue or organ. Targeting cell-specific ER components such as cAMP-responsive element-binding transcription factor H (CREBH) may be a promising strategy. The implementation of nanotherapeutic targeting approaches would be helpful for resolving these problems and providing new advances in efficient prevention of ER stress and treatment of ER stress-related diseases.

Using nanoparticles loaded with a therapeutic agent and coated with a monoclonal antibody against a tissue-specific antigen is a promising strategy for targeted delivery of a drug at high local concentrations. However, the development of nanotherapeutic tools for targeting cardiovascular ER stress-induced apoptosis is still in its infancy. Delie et al. [150] constructed polymeric nanoparticles capable of recognizing the COOH-terminal ER retention domain of GRP78, which is markedly overexpressed in prostate and ovarian cancer. The nanoparticles were able to deliver a cytotoxic agent, paclitaxel, to GRP-78-positive cancer cells. Niu et al. [151] reported a cardioprotective effect of nanoparticles loaded with cerium oxide (CeO_2_), a ROS scavenger, in transgenic mice with cardiac-specific expression of monocyte chemoattractant protein- (MCP-) 1 that causes ischemic cardiomyopathy associated with the activation of ER stress. In heart failure, MCP-1 is involved in cardiomyocyte death through ROS-induced ER stress and apoptosis mediated by MCP-1-induced protein (MCPIP), a proapoptotic transcription factor [152]. CeO_2_ nanoparticles injected intravenously inhibited progressive left ventricular dysfunction and dilatation in MCP mice by reducing oxidative stress and ER stress associated with suppression of expression of key ER-stress-related proteins [151].

The development of therapeutic nanoparticles capable of prolonged circulation in the bloodstream may provide an effective alternative method for treating ER stress in atherosclerosis and other cardiovascular diseases. For example, liposomal encapsulation of a drug and further liposomal pegylation significantly increase drug stability and residence time in blood as well as decreasing its cardiotoxicity [153]. In a rat ischemia/reperfusion model of cardiac injury, Takahama et al. [154] showed significantly advanced cardioprotective properties for prolonged adenosine encapsulated in pegylated liposomes compared to free adenosine. Knowledge regarding the mechanisms of the UPR and ER-stress-related diseases has rapidly accumulated in recent years, but many questions remain unanswered. Investigations of the mechanisms and pharmacological actions of ER stress are important in providing new mechanistic insights and developing novel targets for ER stress-related diseases. We believe that a more deep understanding of ER stress will open promising avenues for the development of clinically useful drugs.

## Figures and Tables

**Table 1:** Therapeutics targeting molecular components of ER stress and ER stress-induced apoptosis.

Drug	Mechanism	Potential indication	Reference
5-Aminoimidazole-4-carboxyamide-1-*β*-D-ribofuranoside (AICAR)	Reduction of ER stress by AMPK activation	Ischemic heart disease, heart failure, cardiac hypertrophy, atherosclerosis	[8–10]

BiP inducer X	Induction of GRP78	Heart failure, stroke	[11, 12]

Curcumin	Induction of GRP94	Heart failure, atherosclerosis, thrombosis, diabetes, diabetic cardiomyopathy, inflammation, dyslipidemia	[13]

CS-866	Reduction of ER stress by pressure-overload	Heart failure, cardiac hypertrophy	[14]

EN460	ERO1*α* inhibitor	Prevention/reduction of ER stress-induced oxidative stress	[15, 16]

Benzodiazepinones	ASK1 inhibitor	Atherosclerosis, cerebrovascular ischemia	[16]

QM295	ERO1*α* inhibitor	Prevention/reduction of ER stress-induced oxidative stress	[15]

Isoproterenol	Proteasome activation and assembly	Heart failure, atherosclerosis	[17]

Pioglitazone	Reduction of ER stress	Heart failure, atherosclerosis	[18]

Phenylbutyrate	Chemical chaperone	Heart failure, atherosclerosis, pulmonary hypertension	[19–21]

Pravastatin	Reduction of ER stress by pressure-overload	Heart failure, cardiac hypertrophy	[22]

Salubrinal	Prevention of eIF2a dephosphorylation	Heart failure, cardiac hypertrophy	[23]

SB203580	CHOP phosphorylation	Heart failure, cardiac hypertrophy, atherosclerosis	[24]

SP600125	Prevention of CHOP induction by stretch	Heart failure, cardiac hypertrophy, atherosclerosis	[24]

Sunitinib	IRE1 activation	Heart failure, atherosclerosis	[25]

Tauroursodeoxycholic acid (TUDCA)	Chemical chaperone	Heart failure, atherosclerosis	[19]

## Abbreviations

AGE: Advanced glycation end-product
AICAR: 5-Aminoimidazole-4-carboxyamide-1-*β*-D-ribofuranoside
AMPK: AMP kinase
Apo: Apolipoprotein
ASK1: Apoptosis signal-regulating kinase-1
ATF: Activating transcription factor
Bcl-2: B cell lymphoma-2
Bim: BH3-only protein
CHOP: C/EBP*α*-homologous protein
CREBH: cAMP-responsive element-binding transcription factor H
CREBP: cAMP-responsive element-binding protein
eIF2*α*: Eukaryotic translation initiation factor 2*α*
EC: Endothelial cell
ER: Endoplasmic reticulum
ERAD: ER-associated degradation
ERO1: ER oxidase 1
GFAT: Glutamine:fructose-6 phosphate amidotransferase
GLP-1: Glucagon-like peptide 1
G6P: Glucosamine-6 phosphate
GRP: Glucose-regulated protein
GSK-3: Glycogen synthase kinase-3
IL: Interleukin
IP3R1: Inositol-1,4,5-trisphosphate receptor-1
IRE1: Inositol requiring protein-1
7-KC: Ketocholesterol
LDL: Low density lipoprotein
Ldlr: LDL receptor
Lp(a): Lipoprotein(a)
MCP-1: Monocyte chemoattractant protein-1
MCPIP: MCP-1-induced protein
PARM-1: Prostate androgen-regulated mucin-like protein 1
PBA: Phenylbutyrate
PERK: Protein kinase RNA-like ER kinase
PDI: Protein disulfide isomerase
PRR: Pattern recognition receptor
RAGE: AGE receptor
ROS: Reactive oxygen species
SERCA: Sarcoplasmic/endoplasmic reticulum Ca^2+^-dependent ATPase
SFA: Saturated fatty acid
SRA: Scavenger receptor
SREBP-2: Sterol response element binding protein-2
TLR: Toll-like receptor
TNF: Tumor necrosis factor
TRAF2: TNF receptor-associated factor 2
TUDCA: Tauroursodeoxycholic acid
UDP-GlcNAc: UDP-N-acetylglucosamine
UPR: Unfolded protein response
VSMC: Vascular smooth muscle cell
XBP1: X-box binding protein 1.
